# Move the north: evaluation of a regional stakeholder engagement initiative to support the development of a community-partnered physical activity research agenda

**DOI:** 10.1186/s40900-019-0167-x

**Published:** 2019-11-27

**Authors:** Chelsea Pelletier, Anne Pousette, Gloria Fox, Robin Keahey, Kirsten Ward, Guy Faulkner, Drona Rasali, Sandra Allison

**Affiliations:** 1 School of Health Sciences, University of Northern British Columbia, Prince George, Canada; 2 Promotion of Wellness in Northern BC (WINBC), Prince George, Canada; 3 Northern Medical Program, University of British Columbia, Prince George, Canada; 4 University Hospital of Northern BC, Northern Health, Prince George, Canada; 5 Population and Public Health, Northern Health, Prince George, Canada; 6 School of Kinesiology, University of British Columbia, Vancouver, Canada; 7 British Columbia Centre for Disease Control, Provincial Health Services Authority, Vancouver, Canada Faculty of Kinesiology and Health Studies, University of Regina, Regina, Canada; 8 School of Population and Public Health, University of British Columbia, Vancouver, Canada

**Keywords:** Community-based participatory research, Population health, Rural health, Physical activity, Knowledge translation, Patient and public involvement

## Abstract

**Background:**

Although it is generally accepted that engaging with members of the public contributes to more actionable and relevant research, there are a limited number of reported evaluations of community engagement initiatives. Certain populations, such as those with lower socioeconomic status and those who live in rural or dispersed communities, tend to face increased barriers to engagement. For researchers and community members alike, it is important to understand and evaluate engagement initiatives to support participatory research methods, particularly when working with underserved or hard to reach populations.

**Methods:**

Over 2-days in October 2018, we hosted a Research Agenda Development Workshop and Physical Activity Summit with relevant researchers, health professionals, and community partners. The objectives of this initiative were to develop a physical activity research agenda based on community-identified priorities, create networking opportunities, and understand factors impacting physical activity participation in communities across northern British Columbia (BC). An evaluation plan was created early in the planning process to understand the reach of the event based on representation targets. Stakeholder satisfaction with the event was evaluated with a post-meeting survey.

**Results:**

The event was successful in engaging community members from a broad geographic region with at least 90 people in attendance from 11 different northern BC communities, representing 46 different organizations. Meeting attendees indicated they were satisfied with the event and felt their perspectives were heard. To advance physical activity in the region, the most commonly desired outcome from the event was the need for ongoing communication channels to support knowledge translation and capacity building in the low-resourced communities of northern BC. There were some gaps in representation targets present at the event. Namely, there were a limited number of people representing Indigenous organizations, and the education and private sectors.

**Conclusions:**

This two-day event was successful at achieving its objectives and engaged a diverse group of stakeholders from a broad geographic region. The outcomes from this event are being used to develop a community-partnered physical activity research agenda and contribute to ongoing learning by the research team to understand contextual factors influencing physical activity in the communities of northern BC. This model of engagement could be used by other researchers interested in engaging with a diverse, multi-sector group of academics, health professionals and community members to support community-centered population health research.

## Plain English Summary

Partnering with community members on research projects helps make research more relevant to them. It is important to understand and evaluate the process of engaging with members of the public to determine if that process has been successful, if the correct people were included, and to provide a guide for other research teams to follow. In this report, we describe an engagement initiative with community members from a broad geographic region, northern British Columbia (BC), Canada, to develop a research program focused on physical activity. This event was held over 2-days and consisted of a Research Agenda Development Workshop and Physical Activity Summit. Combined, there were over 90 participants in attendance from more than 46 different organizations or sectors, representing 11 different northern BC communities. In a post-meeting evaluation survey, people at the meeting indicated they felt their views were heard and that it was a good use of their time. It was also commonly stated that this type of event should lead to sustained partnerships, that it is essential for a report to be circulated to all attendees, and there is a need to establish communication channels for ongoing sharing of information. Our team was able to develop a 5-year program of research based on community-identified priorities and an increased understanding of the factors influencing physical activity in northern BC communities. We hope this report helps other research teams interested in conducting similar large-scale regional community engagement initiatives which are integral to community-centered population health research.

## Background

It is generally accepted that patient and public engagement in health research contributes to more relevant, actionable, impactful, and accountable research, ultimately improving health outcomes [1]. By partnering with a broad range of stakeholders and patients, researchers are better able to understand the contextual and cultural realities of communities, mitigate barriers, and identify community-relevant outcomes [2, 3]. Patient and public engagement in research can occur at various stages of the research process and may include identifying research questions and priorities, participation on advisory councils to guide study development, recruitment, implementation, and knowledge dissemination [4]. A spectrum of engagement is described by the International Association for Public Participation (IAP2) with activities ranging from informing to empowering [5]. Health research funding bodies are increasingly requiring researchers to include aspects of patient and public engagement in research across this spectrum. There are several frameworks to guide this engagement in research, including the Strategy for Patient-Oriented Research (SPOR) Patient Engagement Framework from the Canadian Institutes of Health Research [6] and INVOLVE from the National Institute for Health Research (NIHR) in the United Kingdom [7].

Despite the increased expectation that researchers partner with knowledge users, patients, and the public in knowledge creation, the criteria, process, and outcomes of successful engagement activities are underreported [2, 4, 8]. Patient and public engagement in research is most common in the early stages of research to support agenda setting and priority development, with less engagement at later stages of the research process [4, 8]. There is limited understanding about how to most effectively engage with the public to inform health research and a need for more robust evaluation and reporting of engagement processes [2, 4, 8]. This contributes to barriers for researchers in determining how to engage with members of the public, the costs associated, and uncertainty about identifying what types of activities are worthwhile [9–11]. Concerns about feasibility are also important to consider. Specific concerns include strategies to engage with hard to reach populations and being able to include meaningful engagement within restricted funding cycles and research timelines. By describing the costs, resources, and impacts of engagement, robust evaluations will increase clarity of use by other researchers and provide evidence to encourage ongoing financial support for such endeavors by funding bodies [12, 13]. It is also essential to evaluate and report on best practices for community engagement activities to avoid tokenism and ensure the right perspectives are included, particularly among marginalized or hard to reach groups who face increased barriers to participation [4, 9, 14, 15].

**Fig. 1:**
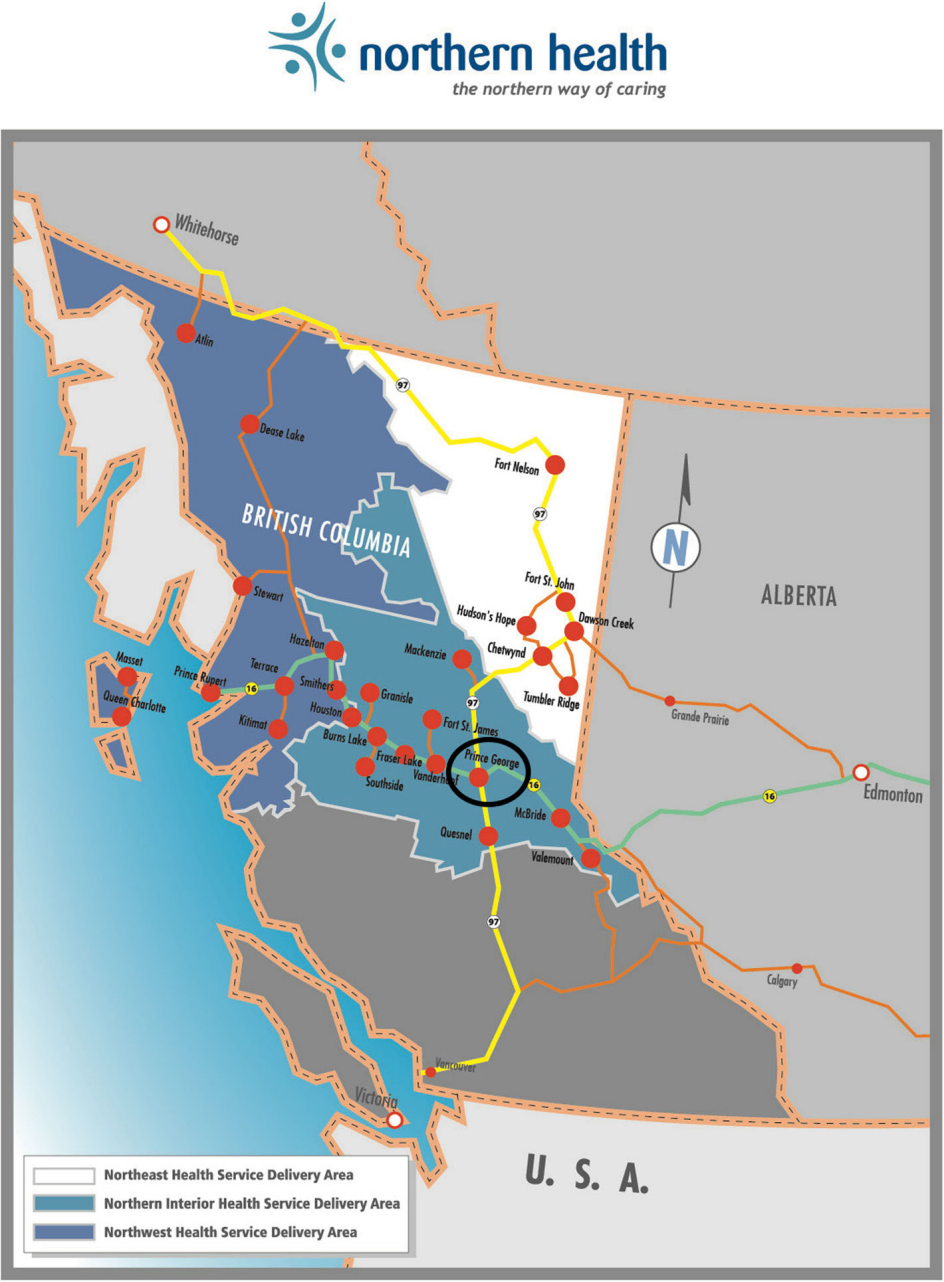
Map of Northern Health region and health service delivery areas. Source: https://www.northernhealth.ca/about-us/quick-facts

The provincial north of British Columbia (BC) covers the northern two-thirds of the province and is diverse in terms of geography, culture, and values. Many of the 32 municipalities and over 127 unincorporated communities of northern BC are considered geographically rural or remote (Fig. 1). This area is served by the Northern Health Authority and is defined as the area north of Quesnel to the Yukon boarder and from the Alberta boarder West to Haida Gwaii (Fig. 1). People living in rural and dispersed regions, such as northern BC, face numerous transportation and sociocultural barriers to research participation. This presents unique challenges in conducting community-centered population health research, where it is necessary to consult with a broad range of individuals to understand the context of program delivery and avoid tokenistic engagement based on convenience.

Northern BC faces a disproportionate burden of noncommunicable disease contributing to worse health outcomes when compared to other regions of the province [16]. This is due to sociocultural and environmental factors that contribute to decreased participation in health promoting behaviors, such as physical activity, and ultimately a decreased life expectancy, common in other rural areas [17, 18]. Recognizing that physical activity is one of the top four strategies for the prevention of noncommunicable disease [19], our research team aims to mobilize a regional physical activity strategy that is supported by a robust and relevant research agenda to advance physical activity in northern BC. Our team recognized early in the process the need to establish authentic relationships with community members in order to incorporate the patient and public voice in our integrated research and knowledge translation approach. Foundational relationship building, pre-formative collaborative work, and knowledge sharing and exchange are key components of the framework for collaborative research proposed by Rycroft-Malone and colleagues [20] and understanding knowledge in the local context is a key element in the Knowledge to Action Cycle of implementation [21].

The purpose of this report is to describe and evaluate our stakeholder engagement process for a two-day event which was designed to support the development of a regional physical activity research agenda and to increase understanding of the factors that shape physical activity behavior in northern BC communities.

## Methods

This stakeholder engagement event was held over 2-days in October 2018 in Prince George, BC, a northern and isolated medium population centre of approximately 78,675 people [22] and the unofficial capital of northern BC. Prince George is located on the traditional unceded territory of the Lheidli T’enneh peoples. The first day was a Research Agenda Development Workshop and focused on identifying research priorities. The second day was a Physical Activity Summit and focused on understanding implementation of physical activity in northern BC by understanding the contextual factors that influence behaviour, program sustainability, and capacity building. In total, there were at least 95 stakeholders engaged over the two-day event, 36 at the Research Agenda Development Workshop (Day 1) and 91 at the Physical Activity Summit (Day 2), with the majority of attendees participating in both days of the event.

The objectives of this initiative were to:

- Identify research priorities, questions and outcomes;
- Determine steps required and key components of a collaborative physical activity research agenda;
- Make connections, network, and bring together people working in various sectors to share ideas about advancing physical activity in the north;
- Understand the application of provincial, national, and international physical activity frameworks in the context of northern BC;
- Clarify next steps needed to bring ideas into fruition – an action plan for northern BC.

### Project team

Our project team includes academic expertise in physical activity and population health (CP, GFaulkner), the non-profit sector (AP), population health surveillance (DR), health care providers (AP, SA), and health systems decision-makers from the regional health authority, Northern Health (SA, GFox). Two research trainees were also engaged in this project (RK, KW). Experts in implementation science and facilitation were invited to contribute and participate in the event.

A core group of 5 working group members (CP, GFox, AP, KW, RK) met regularly to complete pre-meeting tasks, analyze survey results, and prepare meeting documents. The entire research team met monthly or biweekly leading up to the event to identify ‘big picture’ ideas, make final decisions, finalize the agenda, and to approve all event documents. To support the development of the research agenda, our team first completed a scoping review on the implementation of physical activity interventions in rural, remote, and northern communities (manuscript under review).

### Costs and opportunities leveraged

This project was funded by a Convening and Collaborating Award from the Michael Smith Foundation for Health Research (MSFHR C^2^), which provides funding to bring researchers and knowledge users together to plan or co-develop research activities. With this funding in place, we leveraged additional funding from the BC SUPPORT Unit Northern Centre, Northern Health, Wellness in Northern BC (WINBC), and the University of Northern British Columbia (UNBC). This additional funding enabled an expansion of the event and a bursary program to fund community members from across the region to attend the meeting. We were also able to run the event with no registration fee for attendees. In total, we had $23,500 in cash contributions to hold this 2-day event and in-kind contributions of small gifts and draw prizes from local businesses.

### Meeting invitees

Due to funding restrictions and the desire to keep the cost as low as possible for attendees, this event was run primarily as invitation only with no external advertising. Invitees were given the option to indicate other people in their network or organization that should be invited as part of the pre-meeting survey, or the option to send a delegate. To ensure appropriate representation at the event, we created a list of the different lenses (eg, geographic regions, levels of government, community services, not-for-profit special interest groups) we wanted represented at the meeting and brainstormed a list of individuals in different organizations to fit these targets. As invitees registered for the event or responded to invitations, we referred back to our targets and sent purposeful invitations as required to support our diversity targets. These broad categories are described in Table 1.

**Table 1:** Representation targets used to develop the invitation list

Day 1: Research Agenda Development Workshop		
Invited Sector or Diversity Target		Attended
Academic		Yes
Disability/Accessibility/Inclusion		No
Sport & Recreation		Yes
Municipal Government	Planners	No
	Social Policy	Yes
Ministry of Health		Yes
Indigenous		Limited
Early Years & Youth		Limited
Northern Health		Yes
Community Member		Yes
Geographic	Northern Interior	Yes
	Northwest	Yes
	Northeast	Yes
Day 2: Physical Activity Summit		
Invited Sector or Diversity Target		Attended
Academic		Yes
Disability/Accessibility/Inclusion		
	Adapted Activity	Yes
	Chronic Disease	Limited
	New Canadians	Limited
	Low Socioeconomic Status	Yes
	Older Adults	No
Community Service Providers		Yes
Sports & Recreation		Yes
Municipal Government		Yes
Children & Youth	Child Development	Yes
	School District	No
Northern Health		Yes
Provincial	Ministry of Health	Yes
	Not-for-Profit	Yes
Mental Health & Wellness		Yes
Indigenous		Yes
Health Care Professionals		Yes
Community Member		Yes
Geographic	Northern Interior	Yes
	Northwest	Yes
	Northeast	Yes

Note: Limited is used to denote a smaller number of attendees in the respective category than anticipated (e.g. 1 or 2) based on the size of the sector or number of people invited

Save the date notices were sent approximately 4 months prior to the event (June 15, 2018) and full invitations along with a pre-meeting survey and registration were sent approximately 6-weeks prior (September 5, 2018).

### Pre-meeting Survey & Agenda Development

A pre-meeting survey was created for each day of the event to assist in agenda development and to identify breakout group topics. The pre-meeting survey and registration were circulated via email link for completion on the survey platform SurveyMonkey through UNBC Institutional license. Respondents were required to provide consent to disclosing their personal information (name, email) prior to completing the survey to comply with provincial privacy regulations.

#### Bursary program

After completing the registration, participants were given the option to apply for a travel bursary. Anyone was welcome to apply for this support, however, due to a limited amount of funding, it was clearly stated that decisions would be based on identified need and lack of access to additional resources. Funding was offered in the form of reimbursement of travel expenses up to $500. Applications were adjudicated by three team members (CP, GFox, RK).

#### Day 1: research agenda development workshop

In the pre-meeting survey, each attendee was asked to describe their long-term vision for physical activity, and to indicate the contextual factors and cultural norms that impact physical activity in northern BC (see Additional file 1). Responses were analyzed to identify three theme areas: Equity, Cultural Shift, Surveillance & Data. These themes were used to identify breakout groups and the development of the research agenda. During the meeting, attendees were asked to divide into breakout groups and identify research priorities with a timeline for completion. After a list of priorities was created by each group, all workshop attendees were asked to rank the top three priorities in each theme area. The agenda for the Research Workshop is presented in Table 2.

**Table 2:** Research agenda development workshop agenda (Day 1)

Time	Session Topic
8:00–8:30 am	Arrival Continental breakfast
8:30–9:00 am	Welcome & introductions What do you want to get out of today?
9:00–9:10 am	Setting the stage Objectives & ground rules
9:10–9:20 am	History of physical activity in northern BC, 2003–2018
9:20–9:40 am	What do we know about physical activity and health in the north?
9:40–10:00 am	Physical activity policies, strategies, frameworks Provincial, National, International
10:00–10:15 am	Current projects & discussion of concept map
10:15–10:30 am	Health Break
10:30 am – 12:00 pm	Goals and outcomes – vision for 5, 10, 15 years. Small group brainstorming based on key themes identified in survey.
12:00–1:00 pm	Lunch and walk around the ring road
1:00–2:00 pm	Ranking priorities within each of our 3 themes. Given barriers & opportunities, what should our priorities be for each theme?
2:00–3:00 pm	What research questions should we be asking? How do we achieve our vision?
3:00–3:15 pm	Health Break
3:15–4:30 pm	Next steps & summary discussion Who else should be involved in this work? What resources do you need to advance our agenda and where should we seek support? What do you need from a regional physical activity agenda?

#### Day 2: physical activity summit

In brief, attendees were asked: what they would like to get out of the day, the biggest barriers and facilitators to the implementation of physical activity projects in their communities, and to select from a predetermined list of settings (e.g., workplace, schools, primary care, active environments and transportation, sports and recreation) that would be of interest for breakout sessions (see Additional file 2). Responses were analyzed for key themes and used to identify breakout groups and in the creation of a handout and wall posters to support discussions related to implementation. The three breakout groups were based on different physical activity settings: Recreation Centres & Sport, Active Transport & Built Environment, and Health Care.

For the breakout sessions, we pre-identified two leaders and a research trainee to facilitate and take notes during each session, respectively. One of the session facilitators was someone with topic of facilitator expertise and the other was a community physical activity champion known to the project team. Facilitators were also given a handout describing the outcomes, objectives, and goals for each session. Groups were asked to prepare a summary of their discussion for presentation to the larger group. The agenda for the Physical Activity Summit is presented in Table 3.

**Table 3:** Physical Activity Summit Agenda (Day 2)

Time	Session Topic
8:00–8:30 am	Registration
8:30–8:45 am	Introductions & welcome
8:45–9:15 am	Physical activity strategies & concept map Provincial, National, Global
9:15–9:35 am	Sharing Successes – Rapid Fire Session 1
9:40–10:15 am	Breakout groups: Unpacking physical activity strategies for northern BC
10:15–10:30 am	Movement Break
10:30–11:20 am	Keynote Presentation – Choose to Move: Implementation Science in Action
11:20–11:40 am	Sharing Successes – Rapid Fire Session 2
11:45 am – 12:15 pm	Keynote Presentation - A Unifying Vision
12:15–1:00 pm	Lunch
1:00–1:15 pm	Introduction to small group workshops
1:15–2:30 pm	Breakout groups: Working on implementation strategies • Community – Recreation Centres & Sport • Community - Active Transportation & Built Environment • Health Care Settings
2:30–2:45 pm	Summarize & prepare to share group work
2:45–3:00 pm	Movement Break
3:00–3:30 pm	Report back & call to action activity
3:30–3:50 pm	Sharing Successes – Rapid Fire Session 3
4:00–4:20 pm	Bringing it all together Panel Discussion
4:20–4:30 pm	Evaluation Draw & Closing

### Evaluation of stakeholder engagement

A stakeholder evaluation plan was created early in the planning process based on criteria suggested by Esmail et al. [8]. The representativeness of stakeholders in attendance at the event was evaluated using representation targets identified in our invitation list centered on role/occupation, sector involvement, and geography based on health service delivery area (Northwest, Northeast, Northern Interior, see Fig. 1). Stakeholder participation during the meeting and general satisfaction were measured with reflective team notes and a post-meeting survey. The post-meeting survey was adapted from the Public and Patient Engagement Evaluation Tool [23, 24]. This tool was created to be used by a variety of health systems organizations and is designed to measure the integrity of design and process, influence and impact, participatory culture, and collaboration and common purpose of engagement activities [23, 24]. For brevity and applicability to our event, this tool was shortened to a 9-item survey, with responses on a Likert scale from strongly agree to strongly disagree (see Additional file 3 and Additional file 4).

We also asked the following open-ended questions: 1) how would you like the results of your participation in the research workshop/physical activity summit to be used; 2) what was the best thing about the Physical Activity Summit; 3) if we were to hold another Physical Activity Summit would you attend, and if so when would be the best time to schedule it; and 4) please identify one improvement we could make for future events. Open ended questions were analyzed for key themes. Demographic information was not collected from meeting attendees. Meeting participants were asked to complete the survey at the end of the event simultaneous to a door prize draw.

Immediately following the event, team members reflected on the day in a composite notes file which were used for evaluation and to assist in planning future events.

## Results

### Meeting attendees

Of 60 people invited to attend the Research Agenda Development Workshop, 36 people attended. We invited 182 people to attend the physical activity summit and 91 attended. Success at meeting our representation targets are presented in Table 1. There was limited representation from Indigenous organizations, the education sector, the private sector, and new Canadians or immigrant population. Overall, there were at least 46 organizations or municipalities represented and individuals from 11 different northern BC communities, along with provincial representation from Vancouver and Victoria. We were able to support at least 6 community members (some people indicated they were organizing carpools) to attend the meeting from our bursary program and did not deny support to anyone who asked for it.

### Post-meeting evaluation survey

**Fig. 2:**
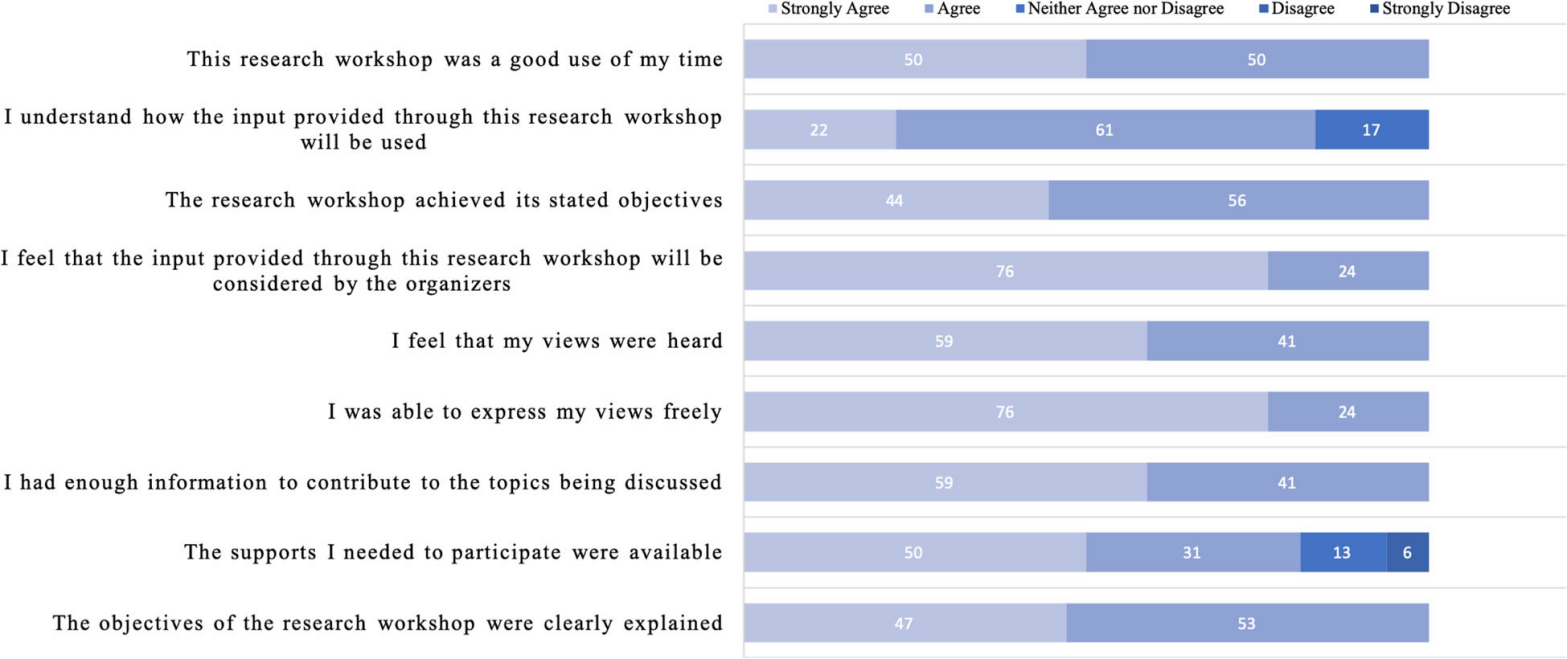
Stakeholder engagement evaluation for Research Agenda Development Workshop (Day 1). Note: Each bar represents % of total responses. Total number of responses = 18 for each question

**Fig. 3:**
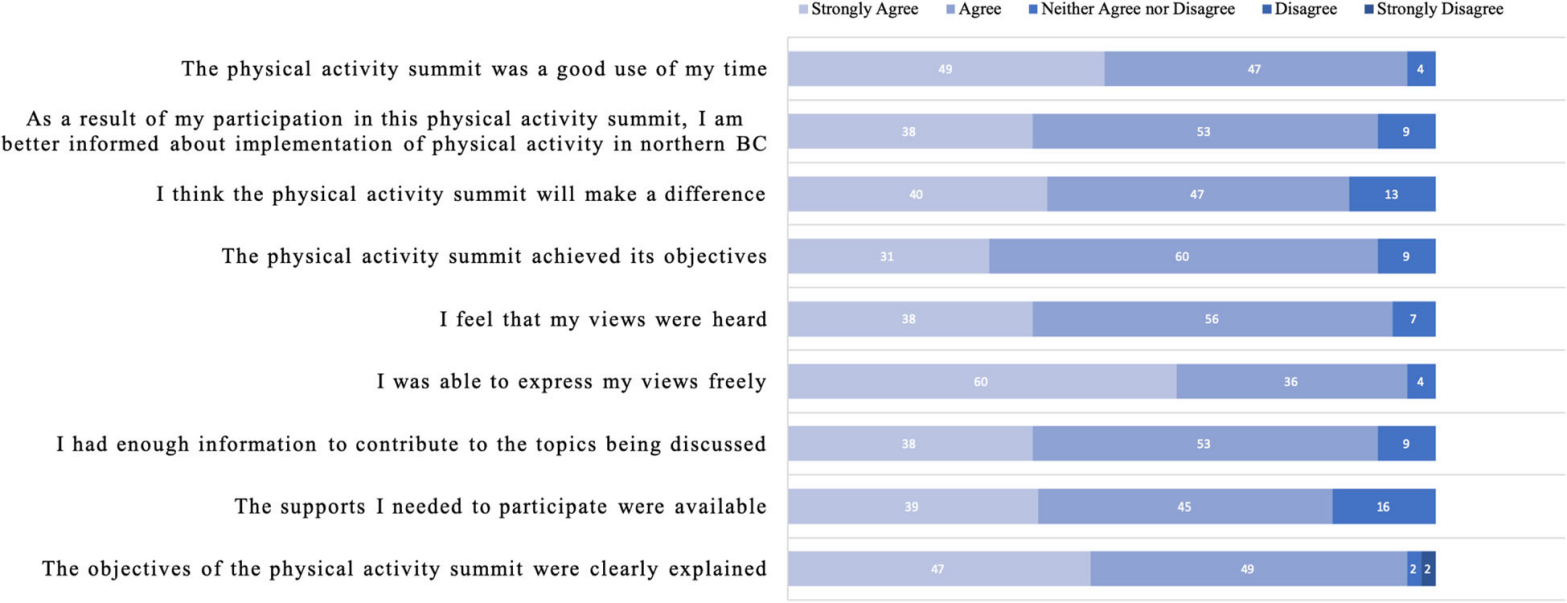
Stakeholder engagement evaluation for Physical Activity Summit (Day 2). Note: Each bar represents % of total responses. Total number of responses = 46 for each question

Responses from the post-meeting evaluation survey are summarized in Fig. 2 (Day 1), Fig. 3 (Day 2), and Table 4 (open-ended questions for both days). There were 18 responses to the Day 1 survey and 46 responses to Day 2. Overall, meeting attendees rated that they either strongly agree or agree with statements regarding participation in the 2-day event. Meeting attendees from both days also indicated that they hoped the results of the event would lead to sustained collaborations and partnerships, and that the results would be used to create change and be shared. Meeting attendees also indicated that we need to continue to strive for greater inclusion, more movement breaks, and to clarify expectations for smaller breakout groups.

**Table 4:** Summary of themes from open-ended questions from post-meeting evaluation survey

*Day 1: Research Agenda Development Workshop*	
**How would you like the results of your participation in the Research Workshop to be used?**	
Guide action	Inform research questions Asking right questions that are important to communities
To support collaboration	Sustained connections Communication – results & process
**Please identify one improvement we could make for future events.**	
Missing perspectives	Indigenous story tellers Have broader inclusion Ask people to suggest others to include in advance
*Day 2: Physical Activity Summit*	
**How would you like the results of your participation in the Physical Activity Summit to be used?**	
Shared	Feedback taken south to decision makers Circulated to all attendees
Change	To move forward Make change Develop future events Increase physical activity rates and decrease inequities
Future partnerships & collaboration	Communication channels Creation of Northern Physical Activity Alliance Information, support & training for all communities to increase capacity
**What was the best thing about the Physical Activity Summit?**	
Idea sharing	Guest speakers & rapid-fire presenters Inspired by what others are doing in their communities
Learning	Recognizing shared challenges Hearing and sharing stories Physical activity actions at different levels
Networking & connections	Meeting potential partners and different stakeholders in physical activity in the north Attempt at positive change that is led by the north
**Please identify one improvement we could make for future events**	
Agenda	Morning was heavy with content More movement breaks Jargon and acronyms used in presentation that were not understood
Logistics and organization	Standardize small group discussion & reporting, clarify guidelines/outcomes Rotate between communities Some key lenses/stakeholders missing – schools, local government Additional travel support and funding

### Meeting outputs

A summary report was created and circulated to all meeting attendees on March 1, 2019. This included a summary of who was in the room and five key action items:

1. The creation of the Northern Physical Activity Alliance to establish communication channels and multi-sector partnerships;
2. Understand the context of physical activity in northern BC communities including the meaning of physical activity, and outcomes that are important to communities;
3. UNBC and Northern Health leaders work together to develop a strategy to build capacity for physical activity, and support community champions and volunteers who are doing this work in their community;
4. Focus on shifting the culture of physical activity to make it part of everyday life;
5. Help to streamline and facilitate community program and infrastructure granting processes, understand how to make the process more accessible, and increase the communication of opportunities.

Research priorities from each of three theme areas were summarized according to a timeline for completion and are presented in Table 5. Meeting attendees were encouraged to send any suggested revisions or feedback on the document, however no feedback was received.

**Table 5:** Research priorities, questions, and projects identified during the Research Agenda Development Workshop based on theme area and timeline for completion

Timeline	Priorities	Research Questions & Projects
EQUITY		
1–2 years	Develop a presence in communities. Understand gaps, barriers, and opportunities. Understand meaning of physical activity to communities. Create central hub or repository of information.	What are the factors that influence physical activity in northern BC communities? What is the northern culture and how does it shape physical activity? What are the inequities that currently exist in physical activity programming between and within northern BC communities?
5 years	Improved knowledge translation, tools, and solutions. Creation of the Northern Physical Activity Alliance.	Understand how to put knowledge to action based on local context and culture.
10 years	Achieve inclusion and integrated approach, where universal access is the new normal.	Development, implementation, and evaluation of inclusion/equity toolkit in northern BC communities.
15 years	Equitable allocation of funding, resources, and research through capacity building.	What are the resources that are needed to achieve universal access in 15 years?
CULTURAL SHIFT		
1–2 years	Defining the problem and issues. Determine what we already know.	What research questions are important to the community? How would the community view success?
5 years	Understand motivators at individual and community levels with northern lens.	What are factors that influence motivation and change at community and individual level?
10 years	Re-evaluation of health statistics – use of data stories to demonstrate effectiveness	What does success look like to rural communities? Reconcile subjective experience with objective data
15 years	Community plans include physical activity targets, infrastructure facilitates physical activity across the life course.	How do we change physical activity beliefs and behaviours in the short, medium, and long term?
SURVEILLANCE & DATA		
1–2 years	Identify data sources and collection tools that are community friendly.	What data is useful at individual, community, and policy level?
5 years	Mobilize community health profiles to include physical activity. Develop framework for measuring outcomes and evaluation of intervention implementation	What data should be collected to inform action?
10 years	Physical activity integrated into ongoing surveillance mechanisms. Data is adaptive and responsive to community priorities.	What are the mechanisms to put data in the hands of people that would apply it effectively?
15 years	Pulling it all together – all data collection points are integrated. Demonstrate rate of progress and alignment with global physical activity strategies.	N/A

## Discussion

This paper describes the engagement of community members in the early stages of research development and presents an evaluation of the reach and effectiveness. Overall, this 2-day community engagement event included a multi-sector group of stakeholders from typically underrepresented communities across a broad geographic region. We were successful at developing a research agenda based on stakeholder-identified priorities and increased our understanding of the context of physical activity in northern BC. Our evaluation has revealed that we were successful at meeting our project objectives to identify research priorities and a community-informed research agenda, although there were gaps in our representation targets.

This community engagement event was designed to support our ongoing research activities and to develop a community-partnered research program. According to the IAP2 Spectrum [5], we consider this event at the level of Involve. This distinction reflects our team working directly with community members, understanding community-specific (or community level) concerns and issues, and seeking feedback on final materials developed based on the engagement activity to confirm contributions were reflected in the final research agenda and steps for action [4]. It is our intention as a research team that the process, outcomes, and relationships developed from this event will support future research projects at the Collaborate or Empower stage of public participation in pursuit of the co-creation of knowledge and implementation of evidence-informed theory-based physical activity interventions [5]. This event serves as a platform to support the development of relationships and networks of community members to engage at different stages and in different projects as part of our overall research program. As part of our commitment to relationship building, we have established a monthly “*Physical Activity Update*” newsletter that is circulated to all meeting attendees and others who have expressed interest in joining our network. This newsletter presents funding opportunities, relevant community events, and provides informational resources to support physical activity in communities. Our team also regularly provides content expertise, supports, and consults on various physical activity related projects in the region to support evaluation and implementation.

At this point, we are unable to evaluate the long-term implications of this event and if it contributes to increased engagement in research projects or ultimately improved health outcomes and service delivery for the region. This event helped our research team to develop a 5-year research program based on community-identified priorities and to establish strong community networks. Moving forward, it is still important to understand and evaluate the process of sustaining these activities through the entire research process. There are many frameworks, strategies, and outcomes proposed for patient and public research engagement [2, 25–27]; however, reports on the process of conducting events, the costs associated, and strategies for broad representation are few. It will also become prudent to evaluate the development of our own diverse research team, as understanding research partnerships between researchers and other stakeholders remains poorly understood and may present an opportunity to close the ‘knowledge-to-practice’ gap [28, 29].

In the post-meeting evaluation, meeting attendees indicated they wanted a formal network of communication and partnership to evolve from this event to support ongoing knowledge translation and for the results to be used and disseminated, not shelved. Community members in attendance also noted the need for support and capacity building with leadership from the health authority and local institutions. This further supports the need for a strong community-university-health authority partnership to formalize relationships and establish communication mechanisms. This approach is becoming more popular in health research and as a health promotion strategy, although the process of establishing these partnerships is substantial and requires mutual trust, respect, effective communication, and a shared vision [26, 30].

Based on our representation targets, we were able to deliver a reasonably inclusive event as nearly every sector in our original plan was included. Because we did not collect demographic information from meeting attendees, only self-reported role and organization, we are unable to make any statements regarding representation in terms of age, ethnicity, culture, or gender. Our Indigenous representation, particularly on Day 1, was limited. For Day 2, we had increased representation from Indigenous organizations. Northern BC includes the greatest relative percentage of Indigenous people in the province, and represents an important stakeholder group for any population health research program. Additional relationship building and collaborative discussion with Indigenous organizations and communities is needed to ensure Indigenous perspectives and way of knowing are included in all future engagement and research activities. Including Indigenous representation on the planning team to ensure an Indigenous lens throughout the project, including in the facilitation of small breakout groups and analysis of meeting outcomes, is suggested for future engagement events. We also had limited participation from the education sector, including early childhood development and youth. There were several local teachers who expressed interest and registered for the event, but were ultimately unable to attend due to difficulties securing teaching coverage for the day. The private sector was also not included in this event. Although not an obvious partner in health promotion research and not without challenges, the private sector does represent a stakeholder group and potential avenues for funding partnerships.

There are also several barriers in the implementation of this initiative to note. At the meeting participant level, these included competing work commitments, travel distance, and cost. We tried to minimize these barriers by running the event in the fall when weather is more appropriate for travel, and hosting the event in a geographically central location. We recognize that there is likely no ideal time or location for everyone. At the organizing team level, barriers to the implementation of this event included competing work obligations, the interpersonal skills required to form relationships, and facilitation skills in conducting the actual event.

### Lessons learned

Based on our team’s reflections and comments from meeting attendees, there are several key takeaways for teams interested in similar community engagement events. The first is the need for proper and sufficient administrative support. Although we had sufficient financial resources to run the event, additional administrative support would have improved logistical aspects of the event. Given the funding restrictions, we were unable to pay a coordinator to assist with planning, and would urge funders to consider this when developing such awards. Another important lesson was the organization of the travel bursary program. This funding was offered based on reimbursement process, in keeping with institutional policies. We realized after the fact that it imposed a barrier on some participants who had to pay the cost upfront before a reimbursement could be processed. Administrative support would also have better supported attendees in booking travel and processing of paperwork.

A second consideration is communication. The majority of communication with attendees was done via email, using an online survey platform for registration. Although this seemed to work fairly well, there were people we were ultimately unable to reach as it relied on our existing networks. A website may have better facilitated the sharing of information and resources, although this would need to be balanced with having the event more open to the general public, in which case a registration fee may have been required. It is also possible that individuals without internet access, which is more common in northern and rural areas, faced disadvantages participating in this event.

Thirdly, identifying background knowledge and developing learning supports for attendees may have helped to make the information presented more accessible to members of the public. Providing a glossary of key terms, common acronyms, and technical jargon are potential strategies for this language barrier. Another option would be to develop a plain language communication guide for all presenters and group facilitators. To gauge learning needs, the pre-meeting survey could include questions about what people would need to support engagement, or terms that we anticipate would be part of the conversation that community members may not be familiar with. Providing training for patient and public research partners, potentially using resources such as those provided in the BC SUPPORT Unit Foundations in Patient-Oriented Research Course [31], may also facilitate participation.

Finally, a higher level of facilitation of breakout groups would have been helpful to ensuring appropriate communication within the group. There was inconsistency in the breakout groups when summarizing or reporting back to the larger group, a template or form to guide reporting may better facilitate activities and enhance quality and documentation of outputs. Other teams may consider the balance between structured sessions and networking/social time throughout the day as community members most commonly reported they valued the networking opportunities of the event.

### Limitations

While the purpose of this project was to actively engage community members in the development of research questions and empower community level activation in physical activity, we did not have a community member representative or patient partner as part of our organizing team. Our organizing team also lacked representation from geographic regions in northern BC outside of Prince George. Inviting a community member to co-facilitate breakout groups may have also added a unique perspective to the event and addressed some identified communication barriers.

Our evaluation is also limited in that we did not establish a mechanism for measuring long-term impacts of this event, although our continued progress toward the creation of a Northern Physical Activity Alliance will enable the ongoing evaluation of our partnership development activities. Our team has plans to host biannual events, which could potentially include asking delegates to reflect on some of these issues and progress made as the partnership evolve. As attendees were not asked to identify their sector or role in the post-meeting survey, we are unable to identify specific engagement outcomes or satisfaction with the event based on our diversity targets or more detailed demographic information.

Finally, we did not attempt to contact individuals who were invited and chose not to attend the event. We did receive a small number of responses from people who were unable to attend due to time conflicts or other work obligations, however this data was not collected in a systematic way and has not been reported. This process would be challenging and likely face a high number of non-responders, but this information may have been useful for researchers planning similar events.

## Conclusions

Based on pre-established diversity targets, this 2-day community engagement event met pre-determined objectives and reached a wide range of community members from a broad and geographically diverse region. Researchers interested in conducting these types of community engagement activities should carefully consider the resource requirements (financial and administrative/staff support) and providing proper supports for community members from all socioeconomic backgrounds and communities to equally participate. This engagement process early in research project planning has enabled our team to develop a program of research that includes community perspectives, and to create a network to serve as a mechanism for ongoing knowledge translation. While this report and consultation process was specific to physical activity, the lessons, steps, and evaluation can be adapted to other population and public health promotion initiatives.

## Supplementary information

**Additional file 1.** Pre-Meeting Survey (Day 1)

**Additional file 2.**  Pre-Meeting Survey (Day 2)

**Additional file 3.** Post-Workshop Stakeholder Engagement Survey (Day 1)

**Additional file 4.** Post-Summit Stakeholder Engagement Survey (Day 2)

## Abbreviations

BC: British Columbia
IAP2: International Association for Public Participation
UNBC: University of Northern British Columbia
