# Supplementary material for: Move the north: evaluation of a regional stakeholder engagement initiative to support the development of a community-partnered physical activity research agenda
Source: Res Involv Engagem. 2019 Nov 27;5:37. doi: 10.1186/s40900-019-0167-x (PMC6882157; doi:10.1186/s40900-019-0167-x)
Supplement: Supplementary file 1 — Additional file 1. Pre-Meeting Survey (Day 1) [file 40900_2019_167_MOESM1_ESM.pdf]

Move the North: Research Agenda Development Workshop (Day 1)

Pre-Meeting Survey

Thank you for your interest in our event, below you will find some background information about the meeting, such as our objectives and a tentative agenda. We would appreciate if you could take 5-10 min to complete the following questions that include your registration to the event and 2 questions to help jumpstart our conversation. We look forward to seeing you at UNBC in October!

The objectives for our Research Agenda Development Workshop are:

- To identify stakeholder-driven research priorities including questions and outcomes
- Determine steps required and key components of a collaborative physical activity research agenda
- Make connections, network, and bring together people working in various sectors to share ideas about advancing physical activity in the north

1. What is your first and last name, affiliation, and role(s) as it pertains to physical activity?

First Name:
Last Name:
Affiliation(s):
Community:
Email:

2. Are you coming to the event on October 25, 8:30 – 4:30, Bentley Centre UNBC
    - Yes
    - No

3. If you are unable to attend, would you like to suggest a suitable replacement from your organization or network?

---

4. How would you best categorize your role or current position?
    - Community member
    - Researcher
    - Municipal government
    - Non-profit organization
    - Health care provider
    - Health service decision maker
    - Physical activity service/program provider
    - Other:

5. With regards to physical activity, where would you like us, as a community and/or region, to be in the next 5, 10, or 15 years?

---

6. What do you see as the unique contextual factors or cultural norms in northern BC as they shape our physical activity behaviour at individual and/or community levels?

7. (Optional) Do you have any dietary restrictions or accessibility requirements you would like to make us aware of?

If you have any additional questions or comments, please feel free to leave them in the space below or contact Chelsea Pelletier, chelsea.pelletier@unbc.ca or 250-960-5383. If you require financial assistance in the form of a travel bursary please indicate this here.
