# Supplementary material for: Move the north: evaluation of a regional stakeholder engagement initiative to support the development of a community-partnered physical activity research agenda
Source: Res Involv Engagem. 2019 Nov 27;5:37. doi: 10.1186/s40900-019-0167-x (PMC6882157; doi:10.1186/s40900-019-0167-x)
Supplement: Supplementary file 2 — Additional file 2. Pre-Meeting Survey (Day 2) [file 40900_2019_167_MOESM2_ESM.pdf]

Move the North: 2018 Physical Activity Summit (Day 2)

Pre-meeting Survey

Thank you for your interest in our event, below you will find some background information about the summit, such as our objectives and a tentative agenda. We would appreciate if you could take 5-10 min to complete the following 10 questions that include your registration to the event and 2 questions to help jumpstart our conversation. Even if you are unable to attend, your contribution by completing this survey will add value to our work and this event. We look forward to seeing you at the Civic Centre in October!

The benefits of physical activity on health and wellbeing are well known. These include mental and physical development, and the prevention and management of chronic disease. Using this knowledge, the goal of our summit is to advance physical activity in northern BC.

The objectives for the Physical Activity Summit are:

- To identify implementation strategies for physical activity in northern BC
- To understand the application of provincial, national, and international physical activity frameworks in the context of northern BC
- To clarify next steps needed to bring ideas into fruition – an action plan for northern BC

1. What is your first and last name, affiliation, and role(s) as it pertains to physical activity?

First Name:
Last Name:
Affiliation(s):
Community:
Email:

2. Are you coming to the event on October 26, 8:30 – 4:30, Prince George Civic Centre
    - Yes
    - No

3. To promote networking and team building, would you like to be listed in a directory of summit attendees to be included in the meeting package?
    - Yes
    - No

4. In terms of your participation in the summit, how would you best categorize your role(s) or current position (may select more than one)?
    - Community member
    - Researcher
    - Municipal government
    - Non-profit organization
    - Health care provider
    - Health service decision maker
    - Physical activity service/program provider
    - Other:

5. Please feel free to identify anyone (including name and contact information) you think should be included and we will send them an invitation.

---

6. What would you like to get out of the day?

---

7. What are some of the biggest barriers preventing the implementation of physical activity projects in your community?

---

8. What are some of the biggest facilitators (or enablers) that can support the implementation of physical activity projects in your community?

---

9. Please provide us with some examples of projects going on in your community related to physical activity.

---

10. Would you be interested in sharing your program successes in the form of a poster/booth/or short video at the summit?
    - Yes
    - No

11. Given our tentative agenda (attached), what settings would you be interested in for our afternoon breakout session? Select up to 3.

- Workplace
- Schools
- Early Childhood Development
- Early Childhood Education
- Community – sports and recreation

- Community – active environments and transportation
- Community centres
- Primary Care
- Secondary/Tertiary Care (e.g. rehabilitation)
- Residential Care
- Other: ________________

12. (Optional) Do you have any dietary restrictions or accessibility requirements you would like to make us aware of?

If you have any additional questions or comments, please feel free to leave them in the space below or contact Chelsea Pelletier, chelsea.pelletier@unbc.ca or 250-960-5383. If you require financial assistance in the form of a travel bursary please indicate this here and we will contact you with more information.
